# Implications for alcohol minimum unit pricing advocacy: What can we learn for public health from UK newsprint coverage of key claim-makers in the policy debate?

**DOI:** 10.1016/j.socscimed.2013.11.041

**Published:** 2014-02

**Authors:** Shona Hilton, Karen Wood, Chris Patterson, Srinivasa Vittal Katikireddi

**Affiliations:** MRC/CSO Social and Public Health Sciences Unit, University of Glasgow, 4 Lilybank Gardens, Glasgow G12 8RZ, United Kingdom

**Keywords:** Minimum unit pricing, Media advocacy, Alcohol, Policies, Newspapers, Newsprint coverage, Media representations

## Abstract

On May 24th 2012, Scotland passed the Alcohol (Minimum Pricing) Bill. Minimum unit pricing (MUP) is an intervention that raises the price of the cheapest alcohol to reduce alcohol consumption and related harms. There is a growing literature on industry's influence in policymaking and media representations of policies, but relatively little about frames used by key claim-makers in the public MUP policy debate. This study elucidates the dynamic interplay between key claim-makers to identify lessons for policy advocacy in the media in the UK and internationally. Content analysis was conducted on 262 articles from seven UK and three Scottish national newspapers between 1st May 2011 and 31st May 2012, retrieved from electronic databases. Advocates' and critics' constructions of the alcohol problem and MUP were examined. Advocates depicted the problem as primarily driven by cheap alcohol and marketing, while critics' constructions focused on youth binge drinkers and dependent drinkers. Advocates justified support by citing the intervention's targeted design, but critics denounced the policy as illegal, likely to encourage illicit trade, unsupported by evidence and likely to be ineffective, while harming the responsible majority, low-income consumers and businesses. Critics' arguments were consistent over time, and single statements often encompassed multiple rationales. This study presents advocates with several important lessons for promoting policies in the media. Firstly, it may be useful to shift focus away from young binge drinkers and heavy drinkers, towards population-level over-consumption. Secondly, advocates might focus on presenting the policy as part of a wider package of alcohol policies. Thirdly, emphasis on the success of recent public health policies could help portray the UK and Scotland as world leaders in tackling culturally embedded health and social problems through policy; highlighting past successes when presenting future policies may be a valuable tactic both within the UK and internationally.

## Highlights

• This study offers the first insights into how key claim-makers have presented arguments about MUP in the media.
• Minimum unit pricing is an intervention that raises the price of the cheapest alcohol to reduce related harms.
• There is a growing literature on the alcohol industry's influence on policy development.
• There is a need to shift focus away from heavy drinkers, towards population-level over-consumption.
• Greater focus should be placed on getting across the message that MUP is part of a wider package of alcohol policies.

## Introduction

Contested policy debates inevitably play out in the news media. Media reporting plays an important role in influencing public opinion of, and willingness to accept, new public health interventions (Gorini, Currie, Spizzichino, Galeone, & Lopez, 2011; Hilton, Hunt, Langan, Bedford, & Petticrew, 2010). On May 24th 2012, legislation was passed by the Scottish Government to become the first country in the world to introduce a minimum price per unit of alcohol. In Scotland in 2009 sales data from the alcohol industry estimated that 11.9 L of pure alcohol were sold per person over the age of 16, with England and Wales considerably lower (9.6 L per person). In units of alcohol this equates to an average weekly consumption of 22.9 units a week for all adults in Scotland (with England and Wales having weekly rates of 18.4 units). With consumption at this level it is unsurprising that Scotland's death rates from chronic liver disease and cirrhosis are around twice as high as those in England and Wales, and (until very recently) were increasing at a time when the rates in many countries were declining http://www.scotland.gov.uk/Publications/2010/08/31093025/13.

There is good evidence of an inverse relationship between alcohol price, consumption levels and related harms (Anderson, Chisholm, & Fuhr, 2009; Booth et al., 2008; Cook & Moore, 2002; Elder et al., 2010; Hagger, Lonsdale, Baggott, Penny, & Bowen, 2011; Rabinovich et al., 2009; Wagenaar, Salois, & Komro, 2009). It is well documented that increased affordability of alcohol over recent decades has been a major factor contributing to the steady rise in drinking and ill health (Rehm et al., 2009; World Health Organization, 2011). Econometric modelling of the impact of minimum unit pricing (MUP) has estimated that a minimum price of 50 per unit of alcohol would increase the price of the cheapest alcoholic drinks, cutting consumption by 5.7% across Scotland (Meng, Hill-McMancus, Brennan, & Meier, 2012; Purshouse, Meng, Rafia, Brennan, & Meier, 2009, pp. 1–193). Importantly, it is a targeted population measure, with the highest costs borne by heavy, high risk drinkers who consume the cheapest alcohol and, in contrast to alcohol duty changes, the police assures price increases are priced on. As with tobacco, alcohol is associated with broader social harms including: increased crime, reduced economic activity and increased economic costs arising from healthcare, policing and prison provision (Babor et al., 2010). Like tobacco, alcohol's burden is highly avoidable.

The strength of evidence for alcohol interventions has resulted in policy debates about alcohol unit pricing. In Scotland two different Bills proposing MUP have been presented to the Scottish Parliament. The first (the Alcohol Etc. (Scotland) Bill) was proposed in November 2009 by the then minority Scottish Nationalist Party (SNP) Government, but failed to gain enough cross-party support and faced robust industry opposition (Holden, Hawkins, & McCambridge, 2012); the second (the Alcohol (Minimum Pricing) Bill) successfully passed into legislation in May 2012 after the SNP majority Government was elected in the 2011 Scottish Parliament general election. The UK Conservative-Liberal Democrat Government announced similar plans to legislate for MUP in March 2012 (HM Government Home Office, 2012). At the time of writing, the introduction of MUP in Scotland is postponed as the legality of the legislation is challenged by the Scotch Whisky Association and the European Commission (Katikireddi & McLean, 2012; Scotch Whisky Association, 2012) and the UK Government's plans for MUP have been put on hold (BBC News Online, 2013).

Although media representations of alcohol have been subject to widespread study, much of this research has focused on advertising (Smith & Foxcroft, 2009) or non-commercial prompts which pervade the symbolic environment through entertainment such as television (Fogarty & Chapman, 2012b; Nicholls, 2011), film (Kulick & Rosenberg, 2001) and music (Herd, 2005; Van den Bulck & Beullens, 2005). To investigate how particular issues are ‘elaborated, contested and redefined’ by key claim-makers in the alcohol policy debate, Hansen and Gunter (2007) offer a framework that draws on communications and political sciences literature. Such a framework has been used to document the substantial influence that corporate actors exert on other areas of public health policymaking, such as tobacco (Durrant, Wakefield, McLeod, Clegg-Smith, & Chapman, 2003; Kline, 2006). Despite a growing literature on the influence of corporate actors in public health policy, there has been relatively little focus on examining the role and influence of key claim-makers in the alcohol industry (Holden et al., 2012), particularly within the current UK policy debate. In recognition, Holden and colleagues conducted an in-depth interview study investigating the different interests of ‘actors’ in the UK alcohol industry, and how they organised themselves to collectively influence MUP policy. They found that while the alcohol industry comprises a range of producers and retailers with diverse interests and some divisions about minimum pricing, they were also able to coordinate their position collectively to lobby where there were advantages in doing so. Similarly, Katikireddi, Bond, and Hilton (2013) interviewed a range of MUP stakeholders (politicians, civil servants, public health advocates, researchers and industry representatives) to investigate the competing ways stakeholders presented the debate in the policy process. They suggest that public health advocates worked hard to redefine the policy issue as primarily a health issue arising from population-level over-consumption, in contrast to critics of MUP who presented it as a social disorder issue among young heavy drinkers. McCambridge and colleagues analysed the claims made by alcohol industry actors in their submissions to the Scottish Government's consultation on MUP. They found that industry representatives highlighted the need to address the alcohol problem, and stressed the importance of an evidence-based approach, but would typically attempt to undermine strong evidence in favour of the intervention, while promoting weak opposing evidence. Further, despite highlighting the need for evidence-based practice, submissions would promote alternative strategies to addressing the problem (such as self-regulation, public information and education) without citing evidence to support them. The authors suggest that including industry voices in policy decisions can be an obstacle to evidence-based policymaking, and advise that policymakers must treat industry representatives' policy positions with caution (McCambridge, Hawkins, & Holden, 2013).

Within the media effects literature, media influences on the public range from informing public opinion and acting as a link between the public and politicians (Torronen, 2003), to essentially setting the agenda, determining which issues command public attention (McCombs, 2005), and how much salience is attributed to issues (Scheufele, 1999). Audiences' readings involve resistance to, as well as alignment with, ideas, and meaning is constructed in a collaborative process between the text and the audience (Burton, 2004). Within the agenda setting paradigm, how details are selectively framed plays an important role in determining public understandings and potential solutions to the problem (Entman, 1993). In this respect, Nicholls (2011) suggests that the media play a role in articulating shared cultural values around alcohol, though Baillie (1996) found that media influence on attitudes about alcohol specifically is either subtle or too complex to be easily measured. At the time of writing, research into public understandings of the MUP policy debate are limited. Findings from a focus group study investigating public opinion concerning MUP found that participants were sceptical about its likely effectiveness in curbing consumption, perceiving it as an unfair intervention punishing those who drink in moderation. Lonsdale, Hardcastle, and Hagger (2012) concluded that there was a failure to recognise the significance of small, incremental reductions in consumption, a preoccupation with the effects of MUP on heavy, dependent drinkers and little evidence to suggest the UK public would support MUP. Such findings may impact the policy agenda and how policymakers perceive public opinions (Nicholls, 2011). Recognition of the need to raise public awareness of a problem while simultaneously creating a climate in which governments are pushed towards advancing policy solutions led Wallack and Dorfman (1996) to suggest a need to address the power gap rather than just the information gap. In this respect media advocacy has been pursued as a strategy for promoting public health policy by using the media strategically to apply pressure for policy change (Wallack & Dorfman, 1996). Inherent in a successful policy advocacy strategy is the need for health advocates to establish themselves as reliable information providers in the ‘climate of opinion’ (Partanen & Montonen, 1988).

This study attempts to map out the dynamic interplay between news media framings from key-claim makers (including industry actors and health advocates) in creating the ‘climate of opinion’ around MUP in the months leading up to the passing of the Scottish legislation. We anticipate that findings from this work will provide useful contributions to the evidence base on how public discourse on public health polices has been constructed through factual news media genres, and may be useful to health policymakers in informing media advocacy strategy for MUP and similar policy interventions within Scotland, the UK and internationally.

## Method

We selected seven UK and three Scottish national newspapers (including their Sunday counterparts) with high circulation figures (National Readership Survey 2012; Newsworks 2012). Publications represented three genres: ‘serious’, mid-market and tabloids. This typology has been used in other newspaper analyses, and represents a range of readership profiles diverse in terms of age, social class and political alignment (Hilton, Patterson, & Teyhan, 2012). Serious newspapers are traditionally broadsheet format, serious in tone, politically diverse and have broadly middle-class readerships. Middle-market newspapers are tabloid format, relatively less serious, and attract older, middle-class, right-wing readers. Tabloid newspapers are less serious, more sensationalist, politically diverse and have broadly working-class readerships. The search period was from 1st May 2011 to 31st May 2012. We selected this time frame to encompass a time period beginning a few days before the SNP formed a majority government (5th May 2011) and ending a few days after the passage of MUP into legislation (May 24th 2012). Relevant articles were identified using the electronic databases Nexis UK and Newsbank, using variations on the search terms ‘alcohol’ and ‘pricing’. All articles were read by one of two researchers (KW, CP) to determine whether they met two inclusion criteria: MUP is the main focus of the article, and the article format is news, commentary or feature (excluding letters from readers). The search identified 302 articles, of which 262 met the inclusion criteria and were eligible for detailed coding and analysis.

To develop a coding frame, researchers (SH and KW) read all the articles and identified categories for coding around *a priori* research questions: who are the key claim-makers offering opinions on MUP; how do they describe the nature and drivers of the current alcohol problem; and what arguments do they offer for and against MUP? All related text for each category was highlighted and typed into frameworks for analysis. SH worked in close collaboration with KW, checking and validating coding in the three frameworks (claim-makers, nature of the problem, and argument for and against MUP). Once all the data were in frameworks, the constant comparative approach (Glaser & Strauss, 1967; Lincoln & Guba, 1985) was adopted to identify patterns and explanations for differences across the data.

## Results

### Sample overview

Between 1st May 2011 and 31st May 2012, 262 news articles about MUP were published in the ten newspapers included in this study. Of these, just under half of the articles (124, 47.3%) were published in UK newspapers, and over half (138, 52.6%) published in Scottish newspapers. More than half of articles (159, 60.7%) were published in ‘serious’ genre publications, with 74 articles published in ‘tabloid’ publications and the remaining 29 (11.1%) in ‘mid-market’ publications (see Table 1). The majority of articles were ‘news’ format (192, 73.2%), with 47 (17.9%) ‘commentary or editorial’ articles and 23 (8.7%) ‘feature’ articles. Across the newspapers, in this time period, there was no clear stance taken either in support or opposition of MUP.

Table 2 details the frequency of citations (either direct quotations or specifically references) of different categories of claim-makers in articles. Politicians, both advocating and opposing MUP, were the category cited most frequently. The key political advocates were Nicola Sturgeon MSP and the SNP Scottish Government. David Cameron, the UK Prime Minister, was also cited as an advocate, which may have led to the policy later being championed by the home secretary Theresa May, though Cameron's health minister, Andrew Lansley, was often cited as a critic. The Scottish Conservatives and Scottish Liberal Democrats followed similar transitions from initial opposition to eventual advocacy of the policy. The election of a majority SNP Government in May 2011, and changes in leadership in both parties, may have precipitated this change in stance. However, there remained critics within the Scottish Conservatives. The main political critics of MUP were the Scottish Labour Party, particularly Dr Richard Simpson and Jackie Baillie. The party were opposed to the policy throughout the period studied, however newspapers highlighted the support of Labour MSP Malcolm Chisholm, a high-profile back-bencher, for MUP.

Alcohol producers were prominent claim-makers and were predominantly critical of MUP. The Scotch Whisky Association and the Wine and Spirit Trade Association were key organisations voicing criticism. Representing a number of alcohol producers, the associations produced high profile, cohesive voices against the policy. However, some producers were advocates; Canadian producer Molson Coors supported MUP, citing their experience of similar policies in Canada.

Public health organisations and charities were united in their support of the minimum unit pricing policy. Figures from the British Medical Association, BMA Scotland and the Royal Colleges were particularly prominent. Academics in the UK and abroad were also cited as advocates, highlighting research evidence to support the policy.

Supermarkets were notable by their absence during the sample period; only Asda and Sainsbury's featured in a few articles. Retail organisations were critical of MUP, particularly the British and Scottish Retail Consortiums and the Scottish Grocers' Federation. Alcohol organisations and charities, such as Alcohol Focus Scotland and Alcohol Concern, were key advocates, however they were not particularly prominent in newspaper articles in this period. The licensed trade, such as pubs, were divided in their support.

### Advocates on the alcohol problem

Advocates of MUP highlighted two dominant drivers of the alcohol problem: cheap alcohol caused by loss leading and irresponsible alcohol marketing. Advocates typically argued that alcohol was available for purchase too cheaply, and linked low prices with loss-leading by supermarkets. One alcohol charity representative stated that there is a: “tsunami of alcohol harm at the moment, caused largely by the huge availability of very cheap alcohol on supermarket shelves” (Guardian, 23rd March 2012), while a senior representative of the British Medical Association (BMA) in Scotland was quoted as saying: “it is simply wrong that alcohol is sold at prices cheaper than fizzy drinks.” (Daily Express, 2nd November 2011). Similarly, a prominent professor of hepatology warned: “people are now dying in their twenties from liver disease and binge-drinking children as young as 12 are falling prey to the ‘pocket-money alcohol business’”. He went on to state: “a minimum price per unit, really tackles cheap, heavily discounted drinks.” (Daily Telegraph, 29th December 2011).

Cheap prices were also associated with pervasive alcohol marketing. An academic made the case that: “governments need to have a clear and unbiased view of the most up-to-date research on alcohol problems and be bolder about tackling some of the root causes such as overly cheap alcohol and irresponsible marketing that encourages heavy drinking” (Herald, 17th May 2012). A alcohol charity representative echoed this: “alcohol has never been more affordable, available or heavily marketed. This is having a huge impact on our young people. Children can't go anywhere without alcohol being promoted on billboards, in the media or on football shirts” (Sunday Mail, 6th September 2011). Another representative of the same charity observed that: “alcohol has become so normalised that our children are growing up in an environment saturated with pro-alcohol messages” (Herald, 1st September 2011).

When describing the alcohol problem, advocates typically did not frame it in terms of over-drinking at the population level. The few quotations that highlighted the population-level problem included Diane Abbott's (Labour MP) statement that “…women's drinking patterns have changed in a generation, reflecting a silent, middle-class epidemic. The problem is not just young ‘ladettes’. These figures reflect the rise of the British ‘Margarita culture’, and some of the surrounding problems. And the easy availability and low price of supermarket booze have led more housewives to drink to excess at home” (Telegraph, 19th May 2012). Similarly, the chief executive of a health think-tank said: “Middle-class drinkers regularly sharing a bottle of wine with dinner are among the risky drinkers unwittingly putting their health at risk by consuming too many units of alcohol each week” (Herald, 30th August 2011). Such quotations from advocates of MUP that might lead readers to perceive the alcohol problem as a broad-reaching rather than confined to small sub-sections of society were infrequent.

### Advocates on MUP policy

There were three dominant arguments expressed by advocates to explain why MUP would be effective in tackling the alcohol problem: It targets cheap drinks and irresponsible retailing; it targets alcohol misuse; and it will reduce social and health harms. Declaring the effectiveness of MUP, an alcohol charity representative stated that: “raising the price of super-cheap drink sold in supermarkets will not penalise moderate drinkers, it will target those who misuse alcohol, and tackle one of the root causes of what is possibly the nation's biggest health and social problem” (Independent on Sunday, 18th December 2011). A Royal College of Physicians (RCoP) representative agreed that: “a minimum price per unit really tackles cheap, heavily discounted drinks” (Telegraph, 29th December 2011). The Herald (9th May 2011) reported that the Association of Police Superintendents supported legislative solutions to cheap alcohol. Theresa May (Conservative MP and Home Secretary) highlighted the targeted nature of the intervention, stating that: “the strategy is targeted explicitly at dangerous drinkers, problem pubs, irresponsible shops and harmful drinks” (Daily Mail, 24th March 2012).

Advocates' support for MUP was often presented in terms of its ability to both improve health and reduce social antisocial behaviour. A senior BMA representative stated that MUP “will reduce the social and health harms associated with excessive alcohol consumption, sadly a trademark of Scotland's national identity” (Scotsman, 10th May 2011), and that “in Scotland at least 8600 alcohol-related hospital admissions and deaths will be prevented” (Daily Record, 15th May 2012). The Telegraph (14th December 2011) reported that a RCoP representative claimed “a 50 p minimum price per unit of alcohol could save nearly 10,000 lives a year”. One alcohol charity representative stated that: “minimum pricing offers us the opportunity to save lives and protect communities from the devastating effects of harmful alcohol use” (The Sun, 2nd November 2011), while another stated: “raising the price of alcohol is one of the most effective ways of reducing alcohol-related harm” (Scotsman 27th January 2012). A spokesperson for a large English brewer stated: “it will help tackle many of the anti-social problems blighting communities” (Sunday Express, 30th October 2011).

Many advocates suggested MUP could change a culture of alcohol misuse. Alison McInness (Liberal Democrat MSP and health spokesperson) stated: “it's a positive and confident step towards changing the culture of excessive drinking in Scotland.” (Sun, 2nd November 2011). The Daily Record (6th October 2011) reported that a senior representative of the Church of Scotland agreed, believing MUP would target Scotland's “deeply troubling relationship with alcohol”. A senior representative of the BMA was quoted as saying “sensible drinking begins with sensible pricing and I hope that minimum pricing will begin the cultural change we need to reduce the alcohol misuse epidemic in Scotland.” (Herald, 29th December 2011). A senior Scottish policeman agreed that legislation might achieve cultural change: “I think we can do something about this. There are examples where society has changed in recent years. Attitudes to drink-driving have changed; also attitudes to smoking and domestic abuse” (Scotsman, 26th January 2012).

The intervention was typically presented in isolation, rather than as part of a broader alcohol policy package. However, a few advocates spoke about MUP as part of a wider strategy. A senior BMA representative made several statements to that end, such as: “A minimum price, as part of a wider strategy, could end Scotland's heavy-drinking culture” (Sun, 15th May 2012), and: “Without addressing price, many of the policies we’ve introduced already will be rendered less effective” (Guardian, 2nd November 2011). Advocates often ‘banded together’ to make a solid case for MUP. In December 2011, many newspapers reported that the Prime Minister had instigated plans to develop a MUP policy (for example Winnett & Mason, 2011). Newspaper coverage surrounding that event described a collective front presented by medical experts. For example, The Scotsman (28th December 2011) reported that: “19 doctors banded together to demand the introduction of a minimum-pricing scheme”, while the Daily Telegraph (28th December 2011) reported that: “a group of 19 leading medical organisations warned that “pocket money prices” for alcohol were endangering thousands of lives every year”.

### Critics on the alcohol problem

There were far fewer statements from critics of MUP on the drivers of the alcohol problem. Critics presented the alcohol problem as created by sub-groups within society, rather than larger structural causes, and used their media interviews to oppose MUP for a broad range of reasons. However, when pressed in interviews they presented two dominant causes of the alcohol problem: problem people (youth binge drinkers and dependent drinkers); and problem attitudes to alcohol. These statements tended to focus on ‘minority’ sub-groups within the population. For instance, Andrew Lansley (Conservative MP and then-Health-Secretary) highlighted the negative behaviours of a minority: “…we have serious problems with both binge drinking and long-term excessive alcohol abuse in a minority of people” (Sunday Herald, 2nd October 2011). A drinks industry trade association representative stated that: “Britain's biggest problem with alcohol is one of attitude and lack of restraint” (Independent, 24th March 2012), while a representative of a national retail trade association suggested that: “tackling irresponsible drinking should not be about legislation, it should be about changing the attitudes to alcohol” (Sunday Herald, 2nd October 2011).

### Critics on MUP policy

They presented varied arguments for why MUP would be a poor solution to the problem, including: it will not be ineffective; it will punish responsible drinkers; it will punish the poor; it will harm businesses; it will lead to illicit alcohol trading; and it will be illegal. While critics typically supported their positions with reasoned arguments, some were quoted as not supporting intervention without presenting explanations. Drinks industry associations described MUP as “misguided” (Telegraph, 25th May 2012) and “wrong” (Express, 7th May 2011), while a free market policy think tank described the “misguided moral crusade” as “anti-fun Victorian paternalism” (Daily Mail, 24th March 2012). Jackie Bailie (Scottish Labour MSP and health spokeswoman) stated that: “we have consistently argued that this policy is the wrong way to tackle this country's alcohol problems” (Daily Record, 14th May 2012).

Most critics included some reasoning for their opposition. Philip Davies (Conservative MP) suggested the prime minister was: “living in cloud cuckoo land”, in believing that MUP could stem antisocial, underage drinking (The Sun, 24th March 2012). The Herald (15th December 2011) reported that: “the Scottish Government's reintroduction of minimum pricing legislation will have the practical effect of raising the price of the cheapest alcohol beyond pocket-money levels but, without changing the culture from the excessive to responsible drinking, there is every likelihood that the murder rate will continue to rise”. This position was echoed by a representatives a drinks industry trade association, who argued: “there is no evidence to prove it will tackle alcohol misuse – in fact, the international evidence suggests problem drinkers are least likely to be deterred by price rises” (Independent 23rd March 2012), and a representative of a retail trade association, who stated that: “…people who abuse alcohol are not sufficiently susceptible to price for it to make any difference” (Telegraph, 5th March 2012).

One drinks industry association was reported as arguing that: “…a minimum price might reduce consumption for normal drinkers, problem drinkers will always rack up the booze in their shopping trolleys, no matter the price” (Scotland on Sunday, 12th February 2012), while another described MUP as “ineffective” (Telegraph, 25th May 2012). A representative of a major international brewer agreed that MUP would be ineffective, arguing that: “to tackle the minority who drink to excess we must make irresponsible drinking socially unacceptable through education, peer or social pressure, parental example and existing laws” (Daily Mail, 30th December 2011).

Some critics backed up their doubts about the intervention's effectiveness, citing a lack of evidence supporting its use. For example, a spokesperson for a major international drinks producer argued that: “…there is no credible evidence from anywhere in the world that it is an effective measure in reducing alcohol-related harm” (Telegraph, 23rd March 2012), and that “…there is no evidence that marginally increasing the price of alcohol has an impact on problem drinkers” (Sunday Telegraph, 1st January 2012).

A common stance taken by critics was that MUP, as a “blunt tool” (Drinks industry association, Telegraph, 5th March 2012), would: “punish the vast majority of responsible consumers” (Drinks industry association, Independent, 16th February 2012) and “penalise the majority for the actions of the few” (supermarket, Independent on Sunday, 18th December 2011). When espousing these views, critics often highlighted the responsible behaviour of the majority. A representative of a civil liberties group suggested that the punishment experienced could be acute: “This measure penalises the responsible majority – the weekly shopping bill goes up and celebrations such as Christmas for all the relatives becomes unaffordable” (The Sun, 24th March 2012). Many characterised the intervention as a tax, such as a retail trade association whose representative stated: “Effectively, a minimum price is a tax on responsible drinkers” (Independent, 24th March 2012).

In addition to penalising responsible drinkers, many critics depicted MUP as likely to punish the poor. The head of a free market policy think tank described MUP as: “…a miserable, Victorian-era measure that explicitly targets the poor and the frugal, leaving the more expensive drinks of the middle classes untouched” (Daily Telegraph, 15th Match 2012). This view was also presented by a drinks industry association, who said of MUP: “It punishes those on limited and fixed incomes” (Daily Express, 7th May 2011).

Another common critique of MUP is that it would harm businesses, in addition to individuals. A drinks industry association representative predicted MUP would: “…adversely affect millions of consumers and businesses in the UK, while doing nothing to tackle the root cause of alcohol misuse” (Independent, 24th March 2012). An economic analyst suggested that: “…if minimum pricing at 50p per unit was introduced throughout Great Britain we would estimate that 2800 jobs would be lost at alcohol producers and their suppliers” (Sunday Telegraph, 1st January 2012). A whisky industry association spokesperson expressed concern “…at the long-term effect on the Scotch whisky industry in our export markets” (The Sun, 11th May 2011), while a pub and bar industry association representative expressed concern for pubs: “A 50p hike in the price of a pint would be another nail in the coffin of Scotland's pubs” (Daily Record, 13th September 2011).

Some critics reasoned that increasing prices at legitimate retailers may result in a growth in black market and cross-border alcohol purchases, which may in turn cause further harm to retailers. The spokesperson of a retail industry group suggested that MUP will increase “…illicit and cross-border sales to the detriment of indigenous Scottish retailers” (Herald, 15th May 2012). A supermarket spokesperson listed the illicit and alternative trades that might develop: “black, gray, counterfeit, internet and cross-border” (Herald, 18th January 2012).

Many critics suggested that a policy containing MUP would be illegal under European law. The Guardian (16th February 2012) reported that “The drinks industry says it is illegal”, while The Sunday Telegraph (15th April 2012) reported that drinks producers and drinks industry trade organisations “believe it will fail comply with European law as it acts as a domestic barrier to free trade” (Sunday Telegraph, 15th April 2012). One drinks industry association were quoted as stating: “minimum pricing is illegal under European laws” (The Sun, 24th March 2012), and another “we believe minimum pricing would be illegal as it would breach EU and international trade rules” (Sunday Mail, 3rd July 2011). The Scotsman (9th February 2012) reported that alcohol industry doubts over the legality of MUP were formed “…after a legal opinion sought by the Swiss government in 2009 concluded that minimum pricing is not compatible with the EU Treaty”.

### Critics presenting arguments against MUP

Two key features of the way critics argued against MUP were identified. Firstly, they often gave quotes with multiple arguments in one statement and, secondly, their arguments about MUP policy were consistent over time. For example, the Herald (8th September 2011) quoted an economist combining multiple arguments in one statement: “the proposal of a minimum price is extremely weak, would not target problem drinkers and could have a negative economic impact in terms of jobs, trade and costs to the consumer”. The Herald (15th May 2012) also quoted a representative of a drinks industry association as saying: “alcohol misuse in Scotland must be tackled, but minimum unit pricing is not the right route. It will be ineffective, it will be ruled illegal, and it will hurt Scotch whisky exports”, while the Scotsman (24th March 2012) quoted a spokesperson of the same organisation as saying: “Minimum unit pricing of alcohol would be ineffective in tackling alcohol misuse, has been ruled illegal elsewhere in Europe, and would damage the Scotch whisky industry in key export markets as well as at home”. By combining several critical messages into one statement, and delivering those arguments consistently over time, critics of MUP presented an organised and potentially persuasive front.

## Discussion

In 1997 Casswell (1997) argued that public debate about alcohol was dominated by mass media representations and concluded that it is essential for public health advocates to contribute to those representations to help achieve their goals. Similarly, Dorfman and Wallack (1998) concluded that advocates must understand modern media's roles in policy debates and actively work as bridges between researchers and journalists to communicate accurate health stories. Case studies have shown that public health advocates can use the media strategically to promote public health and increase public support for health policies (Stewart & Casswell, 1993; Wallack & Dorfman, 1996), and numerous voices have called for greater, and better, use of media advocacy in promoting health-related alcohol policy (Casswell, 1997; Dorfman & Wallack, 1998; Fogarty & Chapman, 2012a, 2012b; Myhre, Saphir, Flora, Howard, & Gonzalez, 2002).

Media advocacy is not straightforward, as mass media act as active (and potentially antagonistic) participants in debates, as well as conduits for stakeholders' views (Shanahan, McBeth, Hathaway, & Arnell, 2008), and while advocates may be able to agree on a strong message to provide to journalists when solicited, they cannot control the context and content of opponents' contributions, and, as Otten (1992) suggests, it is important that journalists report the impact of interest groups on policy. Advocates need to be aware that small nuances in the way an argument is presented can dramatically affect audiences' appraisals (Hansen & Gunter, 2007). In addition, advocates need to consider the political leanings of newspapers and where their policy sympathies may lie. In this policy debate much like the politicians, traditional right-wing and left-wing newspapers tended to represent a range of views and show splits on the reporting of the drivers for the alcohol problem and MUP as one policy solution.

Anderson, do Amaral-Sabadini, Baumberg, Jarl, and Stuckler (2011) describe a range of potential interventions to encourage healthier alcohol use, and discuss strategies for communicating these interventions. They suggest that advocates should communicate both the severity of the various burdens caused by alcohol and the strength of evidence supporting the interventions, while highlighting the ineffectiveness of strategies currently in use. They suggest that successful communication would emphasise the reduction of binge drinking and protecting individuals from the harmful alcohol consumption behaviours of others. However, our analysis suggests a need to also focus on the population-level harms of alcohol consumption in order that people can draw a clear parallel between the population-level risks and need for a population level intervention. Consistent with this idea is Fogarty and Chapman's suggestion, based on their analysis of television coverage of alcohol and health, that suggested a need for advocates to focus on the long-term risks of alcohol misuse, and to describe the evidence and arguments for interventions (Fogarty & Chapman, 2012a). Perhaps more fundamentally, Myhre et al. (2002) found that general reporting on alcohol in American newspapers is rarely framed in any sort of health context, and suggest that advocates should work towards increasing the frequency and improving the framing of alcohol as a health issue in newspaper coverage. As Hansen and Gunter (2007) explain, determining how best to communicate policies is important as policies that aim to restrict specific behaviours are likely to be framed in news coverage as threats to freedom of choice. Formulating a message that describes the extent of the problem and the evidence for the intervention, and presents that intervention as a cost-effective and fair one, may serve to ease public doubts identified in qualitative research into attitudes about MUP (Hagger et al., 2011; Lonsdale et al., 2012)

While media advocacy is complicated, there is evidence to suggest that the media climate has become more favourable to alcohol intervention advocates over time; Lemmens, Vaeth, and Greenfield' (1999) content analysis of American newsprint media between 1985 and 1991 found that reporting on alcohol issues had shifted away from focussing on individual drivers towards presenting alcohol as a structurally-driven public health issue. This shift in focus over time has also been found in news media reporting on obesity (Hilton et al., 2012). If this trend on alcohol reporting was found to continue, it might suggest that alcohol framing is now likely to be more supportive of structural solutions than it has been in the past. Furthermore, there is evidence to suggest that a recently-devolved Scotland provides greater opportunities for policy promotion, due to a more open policy style allowing easier access to policymakers (Cairney, 2011; Holden & Hawkins, 2012). Indeed, a Bill containing a MUP intervention has been successfully passed in the Scottish Parliament, while the issue has not progressed far in its UK Westminster counterpart.

As suggested by (Holden et al., 2012) the alcohol industry comprises a range of producers and retailers with diverse interests and some divisions about MUP. However, this analysis found that they largely coordinated their position to present the arguments to the public. By combining several critical messages into one statement, and delivering those arguments consistently over time, critics of MUP presented an organised and potentially persuasive front. Champion and Chapman's (2005) analysis of Australian newspaper coverage on smoking-free legislation concluded that, while industry sources presented arguments that were out of touch with society, health campaigners succeeded in presenting a consistent argument focussing on the unfairness of continuing not to protect the health of certain workers. The authors suggest that this media advocacy effort played a significant role in bringing about the legislation, showing the value of presenting a strong, consistent message. As found by [Reference to unpublished results removed to preserve author anonymity for peer review], advocates of MUP were aware of this value, and worked hard to redefine the policy issues as primarily a health issue arising from over-consumption at the population level. However, the present study found little emphasis of that characterisation of the issue from advocates in newspaper articles. In contrast to the coordinated position of critics, advocates lack of focus on alcohol as a population-level problem might lead the public to perceive the problem (as presented by critics) as largely confined to small sub-sections of society, and therefore that a targeted intervention might be a more appropriate and fair intervention to tackle the problem. This finding is supported by Lonsdale et al. (2012) focus group study, which investigated public opinion about MUP, and found participants were sceptical about its likely effectiveness in curbing alcohol consumption, and did not recognise the significance of small incremental reductions in alcohol consumption. Our findings also support Lonsdale's conclusion that more must be done to address this issue in order to gain wider public support for MUP.

Comparing advocates' quotes with those of critics highlighted that advocates were more focused on describing the problem and its drivers, rather than discussing the potential benefits of the MUP. There were several instances of medical experts banding together to make a solid, unified case for MUP. Further, the intervention was typically presented in isolation, rather than as part of a broader policy package designed to tackle the alcohol problem, which may have been useful in addressing public concerns. There were far fewer statements from critics of MUP on the drivers of the alcohol problem. Critics focused more on presenting the alcohol problem as an issue created by sub-groups within society, rather than larger structural causes, and they used their media statements to oppose MUP policy for a broad range of reasons. They presented multiple arguments for why MUP would be ineffective and was an unfair policy, penalising the reasonable majority for the behaviour of an irresponsible minority, and they highlighted the fact that no other country had enacted similar interventions, presenting the policy as untried and untested.

These findings present health policymakers with several important lessons for media advocacy strategy for MUP within Scotland, the UK and internationally. Firstly, there is a need to redefine the MUP policy issue primarily as a solution to stem over-consumption at the population level, and move away from a focus on young binge drinkers and dependent, heavy drinkers. MUP may be more likely to gain wider public support if people understand the importance of small, incremental reductions in alcohol consumption and see the potential benefits of the intervention for everyone. Secondly, a greater focus on presenting MUP as part of a broader policy package designed to tackle the alcohol problem may help the public perceive MUP as an integral part of the wider policy solution. Thirdly, a greater emphasis on the success of recent tobacco control policies could serve to portray the UK, and Scotland in particular, as world leader in developing policy solutions to tackle culturally embedded health and social problems (Smith, & Hellowell, 2012).

## Declaration of interest

SH and SVK are both involved in planning an evaluation of the impacts of the introduction of minimum unit pricing of alcohol in Scotland. The authors declare no additional conflicts of interest.

## Funding

KW, CP and SH are funded by the UK Medical Research Council as part of the Understandings and Uses of Public Health Research programme (25605200 68096) at the MRC/CSO Social and Public Health Sciences Unit, University of Glasgow. SVK is funded by the Chief Scientists Office as part of the Evaluation of Social Interventions programme (25605200 68093) at the MRC/CSO Social and Public Health Sciences Unit, University of Glasgow.

## Contributions

SH developed the study design and drafted the manuscript. All authors contributed to the development and refinement of the coding frame. KW and CP coded the articles and, SH and KW analysed the data. SVK and CP critically revised the manuscript.

## Figures and Tables

**Table 1:** Number of articles by region, genre and publication.

Title	Total articles		Front page articles	
	*n*	%	*n*	%
United Kingdom				
*Serious*				
Guardian & The Observer	17	6.5	0	0
Independent & Independent on Sunday	12	4.6	0	0
Daily Telegraph & Sunday Telegraph	27	10.3	5	18.5
*Middle-market*				
Daily Mail & Mail on Sunday	10	3.8	–	–
Express & Sunday Express	19	7.3	1	5.3
*Tabloid*				
Mirror & Sunday Mirror	4	1.5	0	0
The Sun & News of the World	35	13.4	0	0
Scotland				
*Serious*				
The Herald & The Sunday Herald	67	25.6	3	4.5
Scotsman & Scotland on Sunday	36	13.7	4	11.1
*Tabloid*				
Daily Record & Sunday Mail	35	13.4	0	0
Total	262	100	13	5.2

**Table 2:** Advocates and Critics of MUP (number of articles in which mentioned) *n* = 262.

Advocates		Critics	
**Politicians** (Nicola Sturgeon MSP; SNP/Scottish Government; David Cameron MP; Scottish Conservative Party; Scottish Liberal Democrats; Willie Rennie MSP)	185	**Politicians** (Scottish Labour Party; Dr Richard Simpson MSP; Andrew Lansley MP; Scottish Conservative Party; Jackie Baillie MSP; Anne Milton MP; Murdo Fraser MSP)	104
**Alcohol Producers** (C&C Group; Greene King; Molson Coors)	11	**Alcohol Producers** (Scotch Whisky Association; Wine & Spirit Trade Association; Diageo)	85
**Public Health Organisations and Charities (Including academics and clinicians)** (BMA Scotland & BMA; Royal College of Physicians; Royal College of Nursing; Institute of Alcohol Studies; Centre for Addictions Research)	89	**Public Health Organisations and Charities (Including academics and clinicians)**	0
**Police**	29	**Police**	0
**Retailers**	0	**Retailers** (British Retail Consortium; Scottish Grocers' Federation)	27
**Alcohol Organisations** (Alcohol Focus Scotland, Alcohol Concern)	21	**Alcohol Organisations**	0
**Licensed Trade** (Scottish Licensed Trade Association)	11	**Licensed Trade**	6
**Economists**	2	**Economists** (Centre for Economic and Business Research)	9
